# Supplementary material for: Wheat GSPs and Processing Quality Are Affected by Irrigation and Nitrogen through Nitrogen Remobilisation
Source: Foods. 2023 Dec 7;12(24):4407. doi: 10.3390/foods12244407 (PMC10742881; doi:10.3390/foods12244407)
Supplement: Supplementary file 1 [file foods-12-04407-s001.zip › foods-2724388-supplementary.docx]

**Supplementary Information**

**Legends**

**Supplementary Figure S1** Rainfall distribution and the average temperatures in 2018–2019 and 2019–2020 growing season.

**Supplementary Table S1** Levels of GSP and its components under different irrigation and nitrogen application treatments in low soil fertility.

**Supplementary Table S2** Levels of GSP and its components under different irrigation and nitrogen application treatments in high soil fertility.

**Supplementary Table S3** Processing quality parameters under different irrigation and nitrogen application treatments.

**Supplementary Table S4** The soil NO_3_-N content in low and high soil fertility field.

**Supplementary Table S5** DNC value under different irrigation and nitrogen application.

**Supplementary Table S6** Path analysis of affecting factors for wet gluten content.

**Supplementary Table S7** Path analysis of affecting factors for dough stability time.

**Supplementary Table S8** Path analysis of affecting factors for dough water absorption rate.

**Supplementary Table S9** Path analysis of affecting factors for sedimentation value.

**Supplementary Table S10** Wheat GSP contents, grain yield, and GSP yield in low and high soil fertility field.


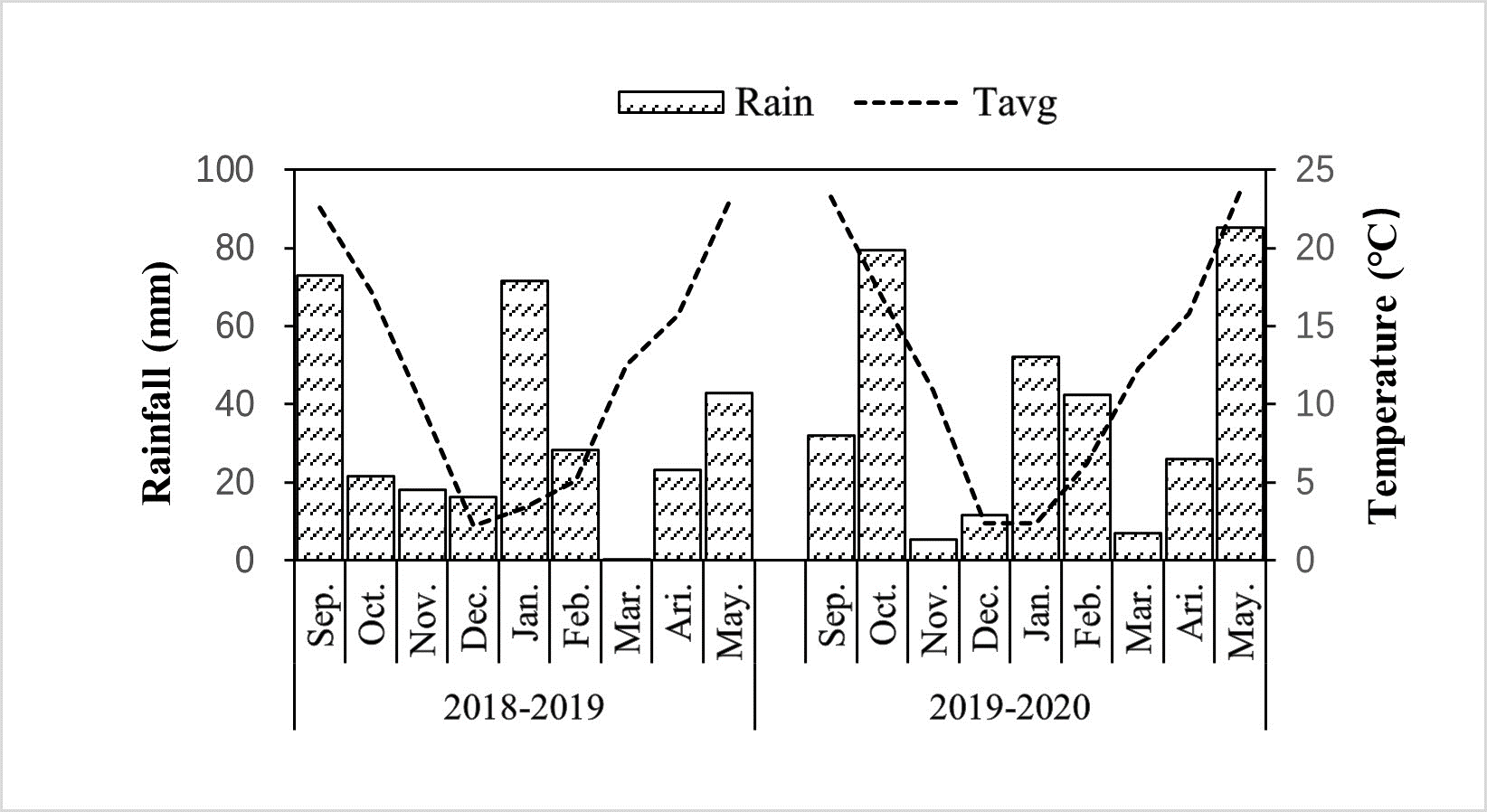


**Supplementary Figure S1** Rainfall distribution and the average temperatures in the 2018-2019 and 2019–2020 growing season. Tavg, average temperature.

| Cultivar | Treatment | GSP | Glutenins | LMW-GS | Ax1 | Bx7 | By8/By9 | Dx5 | Dy10 | Gliadins | ω-gliadin | α/β-gliadin | γ-gliadin |
| --- | --- | --- | --- | --- | --- | --- | --- | --- | --- | --- | --- | --- | --- |
| FDC5 | W0N0 | 53.5 h | 28.2 h | 19.9 f | 1.14 f | 1.90 g | 0.71 f | 2.99 f | 1.47 g | 25.4 i | 0.72 j | 14.3 h | 10.3 g |
|  | W0N180 | 90.4 e | 33.3 g | 23.8 e | 1.44 e | 2.20 f | 0.95 cd | 3.24 e | 1.71 e | 57.1 e | 2.24 g | 33.8 e | 21.1 d |
|  | W0N240 | 99.0 d | 36.1 f | 24.0 de | 1.53 de | 3.17 e | 0.96 cd | 4.33 d | 2.15 d | 62.9 c | 3.82 c | 36.4 c | 22.7 bc |
|  | W0N300 | 106.0 b | 37.8 e | 25.3 c | 1.57 d | 3.19 e | 1.05 ab | 4.33 d | 2.27 c | 68.3 b | 4.30 b | 40.1 b | 23.9 a |
|  | W1N0 | 58.2 g | 29.0 h | 20.0 f | 1.01 g | 2.22 f | 0.91 d | 3.06 ef | 1.68 e | 29.2 h | 0.77 j | 17.3 g | 11.1 g |
|  | W1N180 | 91.6 f | 35.4 f | 23.5 e | 1.50 de | 3.09 e | 0.96 cd | 4.32 d | 2.07 d | 46.5 f | 1.98 h | 26.7 f | 17.5 e |
|  | W1N240 | 100.1 d | 41.2 c | 27.3 b | 1.82 c | 3.69 c | 0.99 bc | 4.85 c | 2.53 b | 58.9 de | 3.05 e | 34.3 de | 21.6 d |
|  | W1N300 | 103.0 c | 43.4 b | 27.6 ab | 2.06 b | 4.05 b | 1.09 a | 5.71 a | 2.89 a | 59.7 d | 3.25 d | 34.6 de | 21.8 cd |
|  | W2N0 | 60.0 g | 28.4 h | 19.8 f | 1.05 fg | 2.08 fg | 0.81 e | 3.06 ef | 1.58 f | 31.8 g | 1.60 i | 17.6 g | 12.6 f |
|  | W2N180 | 99.6 d | 39.4 d | 25.6 c | 1.74 c | 3.55 cd | 0.98 bc | 5.00 bc | 2.51 b | 60.2 d | 2.55 f | 35.6 cd | 22.1 bcd |
|  | W2N240 | 116.0 a | 44.9 a | 28.4 a | 2.17 a | 4.45 a | 1.09 a | 5.84 a | 2.96 a | 71.2 a | 4.72 a | 42.3 a | 24.2 a |
|  | W2N300 | 99.7 d | 38.6 de | 24.7 cd | 1.84 c | 3.47 d | 0.92 cd | 5.21 b | 2.48 b | 61.1 cd | 3.11 de | 35.1 cde | 22.9 b |
| *F* value | Irrigation | 85.5^***^ | 123.5^***^ | 30.2^***^ | 62.6^***^ | 176.2^***^ | 9.8^**^ | 216.4^***^ | 285.7^***^ | 111.6^***^ | 82.0^***^ | 169.0^***^ | 63.4^***^ |
|  | Nitrogen | 1643.5^***^ | 625.7^***^ | 321.7^***^ | 309.2^***^ | 461.7^***^ | 60.7^***^ | 495.8^***^ | 685.2^***^ | 1532.7^***^ | 1166.8^***^ | 1539.2^***^ | 934.4^***^ |
|  | Irrigation X Nitrogen | 44.3^***^ | 43.8^***^ | 25.5^***^ | 29.5^***^ | 39.6^***^ | 12.5^***^ | 41.4^***^ | 61.8^***^ | 41.1^***^ | 42.7^***^ | 89.4^***^ | 13.2^***^ |
| BN207 | W0N0 | 51.9 g | 18.7 i | 12.4 h | 0.80 g | 1.87 h | 1.70 f | 0.66 g | 1.23 f | 33.3 h | 1.46 g | 18.5 f | 13.4 d |
|  | W0N180 | 76.7 e | 22.1 gh | 14.4 g | 1.05 f | 2.36 f | 2.16 e | 0.80 f | 1.36 e | 55.5 fg | 2.99 e | 31.2 de | 20.4 c |
|  | W0N240 | 83.9 d | 25.2 f | 15.5 f | 1.31 de | 2.94 de | 2.62 cd | 1.01 e | 1.75 d | 58.7 de | 3.07 e | 33.6 bcd | 22.1 b |
|  | W0N300 | 91.3 c | 27.4 de | 17.4 d | 1.41 cd | 3.05 cd | 2.71 c | 1.14 d | 1.78 cd | 63.8 b | 4.41 c | 35.3 b | 24.1 a |
|  | W1N0 | 54.0 fg | 21.4 h | 14.1 g | 1.05 f | 2.21 fg | 2.03 e | 0.69 g | 1.29 ef | 32.6 h | 1.41 g | 18.5 f | 12.7 d |
|  | W1N180 | 78.9 e | 26.6 e | 16.5 e | 1.23 e | 3.25 c | 2.69 c | 1.22 bc | 1.77 d | 52.7 g | 2.93 e | 29.4 e | 20.0 c |
|  | W1N240 | 84.4 d | 27.8 cd | 16.9 de | 1.49 bc | 3.46 b | 2.90 b | 1.24 bc | 1.82 cd | 56.6 ef | 3.40 d | 32.3 cd | 20.9 c |
|  | W1N300 | 94.0 b | 31.4 b | 21.1 b | 1.54 b | 2.96 de | 2.57 cd | 1.26 b | 1.94 b | 62.7 bc | 5.17 a | 35.0 b | 22.5 b |
|  | W2N0 | 55.9 f | 22.5 g | 15.4 f | 1.06 f | 2.10 g | 2.00 e | 0.71 g | 1.26 ef | 33.4 h | 2.13 f | 18.8 f | 12.5 d |
|  | W2N180 | 91.3 c | 30.9 b | 21.4 b | 1.32 de | 2.76 e | 2.46 d | 1.13 d | 1.88 bc | 60.3 cd | 4.19 c | 34.0 bc | 22.1 b |
|  | W2N240 | 105.2 a | 37.1 a | 22.8 a | 2.03 a | 4.21 a | 3.68 a | 1.87 a | 2.57 a | 68.0 a | 5.19 a | 37.8 a | 25.1 a |
|  | W2N300 | 89.5 c | 28.8 c | 19.1 c | 1.41 cd | 2.87 de | 2.44 d | 1.20 c | 1.84 bcd | 60.6 cd | 4.81 b | 31.8 cde | 24.1 a |
| *F* value | Irrigation | 140.7^***^ | 346.2^***^ | 341.6^***^ | 67.9^***^ | 49.4^***^ | 37.5^**^ | 311.2^***^ | 106.2^***^ | 27.5^***^ | 5.1^*^ | 106.0^***^ | 25.0^***^ |
|  | Nitrogen | 1304.2^***^ | 423.8^***^ | 241.7^***^ | 166.1^***^ | 232.8^***^ | 201.8^***^ | 748.9^***^ | 278.2^***^ | 710.9^***^ | 272.3^***^ | 414.3^***^ | 477.8^***^ |
|  | Irrigation X Nitrogen | 53.8^***^ | 66.2^***^ | 55.9^***^ | 20.2^***^ | 28.3^***^ | 28.9^***^ | 138.5^***^ | 44.2^***^ | 15.1^***^ | 7.8^***^ | 20.9^***^ | 7.3^***^ |

**Supplementary Table S1** Levels of GSP and its components under different irrigation and nitrogen application treatments in low soil fertility.

10^6^AU/mg, AU represents the peak area of each fraction. FDC5, Fengdecun5; BN207, Bainong207; LMW-GS, low-molecular-weight glutenin subunits. Different lowercase letters indicate significant differences among different treatments within each cultivar (*p* <0.05). *, ** and *** represent significance at *p* <0.05, *p* <0.01, and *p* <0.001, respectively.

| Cultivar | Treatment | GSP | Glutenins | LMW-GS | Ax1 | Bx7 | By8/By9 | Dx5 | Dy10 | Gliadins | ω-gliadin | α/β-gliadin | γ-gliadin |  |
| --- | --- | --- | --- | --- | --- | --- | --- | --- | --- | --- | --- | --- | --- | --- |
| FDC5 | W0N0 | 47.1 i | 24.7 j | 16.6 i | 1.59 f | 2.22 j | 0.27 i | 3.12 j | 0.87 h | 22.4 i | 0.78 g | 13.9 j | 7.7 e |  |
|  | W0N180 | 91.2 c | 30.3 f | 19.7 f | 1.70 cd | 2.96 fg | 0.46 f | 4.04 f | 1.49 f | 60.9 bc | 4.40 b | 37.0 bc | 19.5 a |  |
|  | W0N240 | 96.6 b | 32.4 d | 20.9 e | 1.80 b | 3.29 c | 0.53 e | 4.14 e | 1.64 cde | 62.3 ab | 4.59 ab | 38.0 ab | 19.7 a |  |
|  | W0N300 | 98.0 a | 34.4 c | 21.5 d | 1.90 a | 3.42 b | 0.69 c | 5.06 b | 1.78 b | 63.6 a | 4.76 a | 38.6 a | 20.2 a |  |
|  | W1N0 | 59.7 g | 28.2 i | 18.7 h | 1.62 ef | 2.84 h | 0.40 g | 3.45 i | 1.12 g | 31.5 g | 1.83 f | 19.0 h | 10.7 d |  |
|  | W1N180 | 95.2 b | 35.9 b | 24.3 b | 1.78 b | 3.01 ef | 0.64 d | 4.52 d | 1.63 de | 59.2 c | 4.39 b | 35.3 d | 19.6 a |  |
|  | W1N240 | 99.6 a | 40.1 a | 26.6 a | 1.92 a | 3.54 a | 0.80 a | 5.27 a | 1.88 a | 59.6 c | 3.57 c | 36.2 cd | 19.8 a |  |
|  | W1N300 | 87.6 d | 35.9 b | 23.7 c | 1.71c | 3.32 c | 0.74 b | 4.65 c | 1.70 c | 52.0 de | 3.56 c | 31.6 f | 16.8 b |  |
|  | W2N0 | 52.9 h | 23.9 k | 16.6 i | 1.13 h | 2.30 i | 0.12 j | 3.08 j | 0.63 i | 29.0 h | 1.78 f | 17.4 i | 9.8 d |  |
|  | W2N180 | 74.1 f | 28.6 h | 18.7 h | 1.54 g | 2.90 gh | 0.36 h | 3.57 h | 1.947 f | 45.5 f | 1.84 f | 28.0 g | 15.8 c |  |
|  | W2N240 | 82.6 e | 29.6 g | 19.1 g | 1.66 de | 3.08 de | 0.43 fg | 3.72 g | 1.59 e | 53.1 d | 3.30 d | 32.8 e | 17.0 b |  |
|  | W2N300 | 81.7 e | 30.9 e | 19.7 f | 1.70 cd | 3.13 d | 0.44 fg | 4.20 e | 1.68 cd | 50.8 e | 2.43 e | 31.4 f | 16.7 b |  |
| *F* value | Irrigation | 450.1^***^ | 6815.5^***^ | 4650.5^***^ | 323.8^***^ | 146.2^***^ | 445.0^***^ | 803.3^***^ | 158.9^***^ | 168.7^***^ | 172.6^***^ | 311.6^***^ | 54.1^***^ |  |
|  | Nitrogen | 2537.7^***^ | 6580.8^***^ | 2684.7^***^ | 303.9^***^ | 657.7^***^ | 357.4^***^ | 1330.8^***^ | 1336.1^***^ | 1614.4^***^ | 1908.7^***^ | 613.9^***^ | 657.1^***^ |  |
|  | Irrigation X Nitrogen | 111.3^***^ | 394.5^***^ | 290.2^***^ | 50.5^***^ | 38.0^***^ | 11.5^***^ | 156.1^***^ | 33.8^***^ | 86.4^***^ | 88.6^***^ | 141.5^***^ | 31.2^***^ |  |
| BN207 | W0N0 | 36.3 f | 14.1 g | 9.5 f | 0.54 g | 1.41 f | 1.50 f | 0.36 h | 0.79 f | 22.1 h | 1.49 f | 10.7 f | 9.9 f |  |
|  | W0N180 | 60.8 e | 19.4 e | 11.6 e | 0.94 e | 2.33 d | 2.27 d | 0.83 e | 1.47 d | 41.4 f | 3.34 d | 25.4 d | 12.7 e |  |
|  | W0N240 | 76.2 b | 27.0 b | 16.9 b | 1.21 b | 2.96 a | 2.80 ab | 1.20 b | 1.87 a | 49.2 d | 4.00 c | 27.9 c | 17.3 c |  |
|  | W0N300 | 89.2 a | 32.5 a | 22.0 a | 1.30 a | 3.08 a | 2.89 a | 1.28 a | 1.89 a | 56.7 b | 5.74 b | 32.5 b | 18.4 b |  |
|  | W1N0 | 37.1 f | 18.8 ef | 12.9 d | 0.67 f | 1.81 e | 1.76 e | 0.57 g | 1.02 e | 18.4 i | 1.46 f | 10.7 f | 6.3 g |  |
|  | W1N180 | 68.9 c | 23.3 c | 14.7 c | 1.02 cd | 2.55 c | 2.54 c | 0.93 d | 1.53 cd | 45.6 e | 4.06 c | 27.7 c | 13.8 d |  |
|  | W1N240 | 87.3 a | 26.8 b | 16.9 b | 1.15 b | 2.91 ab | 2.87 a | 1.09 c | 1.81 a | 60.5 a | 6.08 a | 34.4 a | 20.1 a |  |
|  | W1N300 | 76.4 b | 24.3 c | 15.0 c | 1.07 c | 2.75 b | 2.70 b | 1.08 c | 1.68 b | 52.1 c | 4.15 c | 30.8 b | 17.2 c |  |
|  | W2N0 | 32.2 g | 17.8 f | 12.3 de | 0.65 f | 1.76 e | 1.69 e | 0.41 h | 0.92 e | 14.4 j | 1.08 g | 8.1 g | 5.2 h |  |
|  | W2N180 | 59.3 e | 22.2 d | 14.2 c | 0.95 de | 2.41 cd | 2.46 c | 0.70 f | 1.43 d | 37.0 g | 2.37 e | 21.6 e | 13.1 de |  |
|  | W2N240 | 63.7 d | 23.3 c | 14.9 c | 0.99 cde | 2.45 cd | 2.52 c | 0.91 d | 1.50 cd | 40.4 f | 3.96 c | 22.7 e | 13.8 d |  |
|  | W2N300 | 69.3 c | 23.8 c | 14.9 c | 1.04 c | 2.56 c | 2.69 b | 0.97 d | 1.59 bc | 45.5 e | 3.98 c | 27.5 c | 14.0 d |  |
| *F* value | Irrigation | 290.5^***^ | 25.0^***^ | 10.2^**^ | 15.1^***^ | 12.9^***^ | 8.8^**^ | 68.3^***^ | 21.0^***^ | 227.4^***^ | 103.5^***^ | 120.0^***^ | 103.6^***^ |  |
|  | Nitrogen | 2289.9^***^ | 510.1^***^ | 210.8^***^ | 311.8^***^ | 228.9^***^ | 383.1^***^ | 498.3^***^ | 333.2^***^ | 1461.2^***^ | 728.2^***^ | 693.4^***^ | 545.8^***^ |  |
|  | Irrigation X Nitrogen | 82.6^***^ | 97.9^***^ | 80.2^***^ | 18.9^***^ | 14.6^***^ | 10.5^***^ | 18.9^***^ | 12.5^***^ | 39.4^***^ | 14.7^***^ | 60.2^***^ | 29.2^***^ |  |

**Supplementary Table S2** Levels of GSP and its components under different irrigation and nitrogen application treatments in high soil fertility.

10^6^AU/mg, AU represents the peak area of each fraction. FDC5, Fengdecun5; BN207, Bainong207; LMW-GS, low-molecular-weight glutenin subunits. Different lowercase letters indicate significant differences among different treatments within each cultivar (*p* <0.05). *, ** and *** represent significance at *p* <0.05, *p* <0.01, and *p* <0.001, respectively.

**Supplementary Table S3** Processing quality parameters under different irrigation and nitrogen application treatments.

| Soil fertility | Irrigation | Nitrogen | WGC (%) | | DST (min) | | DWR (%) | | SV (ml) | |
| --- | --- | --- | --- | --- | --- | --- | --- | --- | --- | --- |
|  |  |  | FDC5 | BN207 | FDC5 | BN207 | FDC5 | BN207 | FDC5 | BN207 |
| LF | W0 | N0 | 20.3 j | 20.5 g | 8.9 j | 4.7 h | 59.4 e | 59.2 j | 21.0 k | 14.4 j |
|  |  | N180 | 30.9 h | 23.1 f | 9.2 ij | 7.3 e | 62.5 d | 63.1 f | 34.9 f | 30.7 g |
|  |  | N240 | 32.9 fg | 27.6 e | 12.4 f | 7.3 e | 66.3 a | 64.1 c | 39.4 d | 34.2 e |
|  |  | N300 | 35.3 d | 29.9 d | 14.9 c | 7.4 de | 65.8 a | 63.3 e | 41.0 b | 37.7 c |
|  | W1 | N0 | 21.1 ij | 23.3 f | 9.3 hi | 6.1 g | 59.4 e | 59.8 i | 25.6 i | 20.4 h |
|  |  | N180 | 32.1 g | 24.0 f | 10.2 g | 7.5 cd | 62.1 d | 62.8 g | 28.7 h | 32.2 f |
|  |  | N240 | 33.6 ef | 31.4 c | 12.6 f | 7.7 c | 65.1 b | 63.5 d | 34.4 g | 40.2 b |
|  |  | N300 | 41.1 b | 32.2 bc | 16.4 b | 8.2 b | 64.2 c | 62.7 g | 39.0 e | 35.2 d |
|  | W2 | N0 | 21.9 i | 23.4 f | 9.5 h | 6.6 f | 58.2 f | 59.9 h | 21.8 j | 17.0 i |
|  |  | N180 | 34.3 de | 28.2 e | 13.7 e | 8.0 b | 63.6 c | 64.5 b | 39.0 e | 39.8 b |
|  |  | N240 | 43.3 a | 33.8 a | 18.4 a | 8.4 a | 66.6 a | 64.9 a | 41.4 a | 43.9 a |
|  |  | N300 | 37.8 c | 33.0 ab | 14.2 d | 8.2 b | 66.0 a | 63.6 d | 40.3 c | 38.0 c |
| *F* value | Irrigation | | 115.0^***^ | 158.9^***^ | 746.2^***^ | 378.2^***^ | 14.6^***^ | 1185.9^***^ | 1844.6^***^ | 1082.1^***^ |
|  | Nitrogen | | 1017.1^***^ | 515.9^***^ | 2437.5^***^ | 830.8^***^ | 432.6^***^ | 13011.5^***^ | 24172.7^***^ | 10858.3^***^ |
|  | Irrigation X Nitrogen | | 49.1^***^ | 10.1^***^ | 397.0^***^ | 32.0^***^ | 8.1^***^ | 185.2^***^ | 1466.1^***^ | 354.4^***^ |
| HF | W0 | N0 | 18.5 i | 17.4 i | 10.0 h | 4.3 h | 60.2 j | 55.2 j | 21.8 h | 13.0 k |
|  |  | N180 | 26.7 e | 27.4 e | 13.2 e | 4.4 h | 61.3 h | 59.2 d | 30.5 cd | 22.9 h |
|  |  | N240 | 28.8 d | 29.8 bc | 14.3 d | 7.0 c | 62.6 e | 59.8 b | 31.1 cd | 25.3 ef |
|  |  | N300 | 32.0 b | 32.7 a | 16.6 b | 7.9 a | 64.1 b | 60.3 a | 35.1 b | 29.1 a |
|  | W1 | N0 | 19.5 h | 18.5 h | 11.0 g | 5.4 f | 61.3 h | 56.4 h | 23.3 g | 14.8 i |
|  |  | N180 | 29.4 cd | 26.4 f | 14.1 d | 5.8 e | 63.5 c | 58.8 e | 31.8 c | 24.7 fg |
|  |  | N240 | 34.0 a | 30.4 b | 18.4 a | 7.5 b | 65.0 a | 59.8 b | 39.1 a | 27.8 b |
|  |  | N300 | 30.0 c | 29.5 c | 15.4 c | 6.8 c | 63.1 d | 59.5 c | 34.8 b | 26.7 c |
|  | W2 | N0 | 17.6 j | 18.3 h | 10.6 g | 4.4 h | 60.6 i | 55.8 i | 20.8 h | 14.0 j |
|  |  | N180 | 23.5 g | 24.3 g | 12.5 f | 4.8 g | 61.7 g | 58.0 g | 26.5 f | 24.1 g |
|  |  | N240 | 24.8 f | 27.5 e | 14.3 d | 5.7 e | 62.2 f | 58.4 f | 28.7 e | 25.7 de |
|  |  | N300 | 26.5 e | 28.7 d | 15.1 c | 6.4 d | 61.2 h | 58.8 e | 30.0 de | 26.1 cd |
| *F* value | Irrigation | | 1313.2^***^ | 182.8^***^ | 222.8^***^ | 312.3^***^ | 717.5^***^ | 1335.2^***^ | 442.5^***^ | 63.8^***^ |
|  | Nitrogen | | 3798.2^***^ | 5541.2^***^ | 1432.6^***^ | 1236.0^***^ | 876.4^***^ | 13737.2^***^ | 1111.0^***^ | 5591.1^***^ |
|  | Irrigation X Nitrogen | | 184.4^***^ | 78.8^***^ | 112.1^***^ | 125.8^***^ | 200.1^***^ | 506.8^***^ | 62.0^***^ | 78.3^***^ |

Values are annual averages for 2018−2019 and 2019−2020. LF, low soil fertility; HF, high soil fertility; FDC5, Fengdecun5; BN207, Bainong207; WGC, wet gluten content; DST, dough stability time; DWR, dough water absorption rate; SV, sedimentation value. Different lowercase letters indicate significant differences among different treatments within each cultivar (*p* <0.05). *, ** and *** represent significance at *p* <0.05, *p* <0.01, and *p* <0.001, respectively.

**Supplementary Table S4** The soil NO_3_-N content in low and high soil fertility.

| Treatment | LF | |  | HF | |
| --- | --- | --- | --- | --- | --- |
|  | FDC5 | BN207 |  | FDC5 | BN207 |
| W0N0 | 1.54 | 1.17 |  | 3.88 | 2.84 |
| W0N180 | 5.2 | 4.42 |  | 14.44 | 12.51 |
| W0N240 | 7.83 | 6.23 |  | 22.05 | 19.24 |
| W0N300 | 14.43 | 13.22 |  | 24.32 | 21.52 |
| W1N0 | 1.12 | 1.23 |  | 3.31 | 2.57 |
| W1N180 | 2.37 | 2.18 |  | 13.65 | 11.68 |
| W1N240 | 6.29 | 5.97 |  | 20.16 | 17.32 |
| W1N300 | 10.81 | 10.11 |  | 22.17 | 19.47 |
| W2N0 | 0.78 | 0.86 |  | 2.23 | 1.55 |
| W2N180 | 1.87 | 1.66 |  | 14.21 | 10.36 |
| W2N240 | 3.05 | 2.64 |  | 18.56 | 14.38 |
| W2N300 | 9.03 | 8.07 |  | 16.89 | 15.91 |

The soil NO_3_-N content (mg·kg^-1^) were shown the annual average values of 2018–2019 and 2019–2020. LF, low soil fertility; HF, high soil fertility; FDC5, Fengdecun5; BN207, Bainong207.

**Supplementary Table S5** DNC value under different irrigation and nitrogen application.

| Soil fertility | Treatment | FDC5 | | |  | BN207 | | |
| --- | --- | --- | --- | --- | --- | --- | --- | --- |
|  |  | leaves | Stem with sheath | Spike |  | leaves | Stem with sheath | Spike |
| LF | W0N0 | 15.96 | 2.45 | 4.37 |  | 14.45 | 1.84 | 5.02 |
|  | W0N180 | 28.64 | 3.01 | 6.47 |  | 27.18 | 1.95 | 8.85 |
|  | W0N240 | 26.7 | 2.33 | 8.66 |  | 28.67 | 3.08 | 7.86 |
|  | W0N300 | 30.33 | 4.49 | 6.03 |  | 28.83 | 4.18 | 6.08 |
|  | W1N0 | 20.2 | 1.83 | 4.87 |  | 21.45 | 1.57 | 3.24 |
|  | W1N180 | 31.28 | 5.11 | 2.87 |  | 33.15 | 3.5 | 2.76 |
|  | W1N240 | 32.82 | 3.17 | 6.9 |  | 35.81 | 2.77 | 9.23 |
|  | W1N300 | 28.93 | 1.49 | 9.36 |  | 34.35 | 2.7 | 10.17 |
|  | W2N0 | 19.01 | 1.46 | 5.94 |  | 22.14 | 1.84 | 5.98 |
|  | W2N180 | 37.18 | 4.46 | 3.39 |  | 41.59 | 4.17 | 5.13 |
|  | W2N240 | 33.77 | 2.97 | 9.63 |  | 40.22 | 2.82 | 6.51 |
|  | W2N300 | 34.45 | 2.38 | 6.26 |  | 39.43 | 4.77 | 5.38 |
| HF | W0N0 | 23.26 | 1.47 | 6.2 |  | 28.15 | 1.46 | 4.62 |
|  | W0N180 | 29.52 | 2.54 | 6.19 |  | 33.77 | 3.04 | 6.27 |
|  | W0N240 | 34.28 | 3.22 | 7.14 |  | 38.05 | 3.18 | 7.5 |
|  | W0N300 | 32.11 | 4.06 | 8.96 |  | 39.35 | 2.6 | 5.71 |
|  | W1N0 | 26.65 | 1.59 | 3.55 |  | 29.07 | 2.03 | 6.89 |
|  | W1N180 | 31.78 | 2.14 | 4.87 |  | 32.18 | 3.31 | 8.21 |
|  | W1N240 | 33.96 | 3.08 | 6.29 |  | 37.78 | 2.81 | 7.57 |
|  | W1N300 | 36.48 | 2.88 | 5.44 |  | 35.67 | 3.37 | 4.38 |
|  | W2N0 | 24.45 | 1.89 | 5 |  | 26.34 | 2.49 | 4.2 |
|  | W2N180 | 27.72 | 3.34 | 6.02 |  | 29.28 | 3.5 | 8.77 |
|  | W2N240 | 30.67 | 2.96 | 7.19 |  | 31.06 | 3 | 9.51 |
|  | W2N300 | 30.99 | 2.92 | 7.05 |  | 30.88 | 3.26 | 7.67 |

The DNC values were shown the annual average values of 2018–2019 and 2019–2020. DNC, the difference in nitrogen concentration for vegetative organs between anthesis and maturity; LF, low soil fertility; HF, high soil fertility; FDC5, Fengdecun5; BN207, Bainong207.

**Supplementary Table S6** Path analysis of affecting factors for wet gluten content.

| Index | Simple correlation coefficient | Direct path coefficient |  | Indirect path coefficient | | | | | | | |
| --- | --- | --- | --- | --- | --- | --- | --- | --- | --- | --- | --- |
|  |  |  | LMW-GS | Ax1 | Bx7 | Dx5 | Dy10 | ω-gliadin | α/β-gliadin | γ-gliadin | Total |
| LMW-GS | 0.705 | 0.188 | 0.000 | -0.037 | -0.240 | 0.031 | 0.457 | 0.079 | 0.211 | 0.017 | 0.519 |
| Ax1 | 0.63 | -0.043 | 0.160 | 0.000 | -0.279 | 0.030 | 0.406 | 0.102 | 0.238 | 0.018 | 0.675 |
| Bx7 | 0.736 | -0.317 | 0.142 | -0.038 | 0.000 | 0.023 | 0.495 | 0.148 | 0.264 | 0.020 | 1.053 |
| Dx5 | 0.443 | 0.037 | 0.159 | -0.035 | -0.194 | 0.000 | 0.307 | 0.012 | 0.149 | 0.009 | 0.408 |
| Dy10 | 0.84 | 0.584 | 0.147 | -0.030 | -0.268 | 0.019 | 0.000 | 0.126 | 0.241 | 0.021 | 0.257 |
| ω-gliadin | 0.667 | 0.245 | 0.061 | -0.018 | -0.191 | 0.002 | 0.301 | 0.000 | 0.249 | 0.019 | 0.422 |
| α/β-gliadin | 0.824 | 0.316 | 0.126 | -0.032 | -0.264 | 0.018 | 0.446 | 0.193 | 0.000 | 0.024 | 0.509 |
| γ-gliadin | 0.82 | 0.026 | 0.120 | -0.029 | -0.248 | 0.013 | 0.468 | 0.175 | 0.295 | 0.000 | 0.794 |

LMW-GS, low -molecular weight glutenin subunits.

**Supplementary Table S7** Path analysis of affecting factors for dough stability time.

| Index | Simple correlation coefficient | Direct path coefficient | Indirect path coefficient | | | | | | | |
| --- | --- | --- | --- | --- | --- | --- | --- | --- | --- | --- |
|  |  |  | LMW-GS | Ax1 | Bx7 | Dx5 | Dy10 | α/β-gliadin | γ-gliadin | Total |
| LMW-GS | 0.795 | 0.263 | 0.000 | 0.073 | -0.095 | 0.596 | -0.316 | 0.256 | 0.019 | 0.533 |
| Ax1 | 0.798 | 0.086 | 0.224 | 0.000 | -0.111 | 0.572 | -0.281 | 0.289 | 0.020 | 0.713 |
| Bx7 | 0.58 | -0.126 | 0.199 | 0.076 | 0.000 | 0.432 | -0.342 | 0.319 | 0.023 | 0.707 |
| Dx5 | 0.898 | 0.704 | 0.223 | 0.070 | -0.077 | 0.000 | -0.213 | 0.181 | 0.011 | 0.195 |
| Dy10 | 0.44 | -0.404 | 0.206 | 0.060 | -0.107 | 0.370 | 0.000 | 0.292 | 0.024 | 0.846 |
| α/β-gliadin | 0.57 | 0.383 | 0.176 | 0.065 | -0.105 | 0.333 | -0.308 | 0.000 | 0.028 | 0.188 |
| γ-gliadin | 0.439 | 0.03 | 0.168 | 0.058 | -0.098 | 0.249 | -0.324 | 0.357 | 0.000 | 0.410 |

LMW-GS, low-molecular weight glutenin subunits.

**Supplementary Table S8** Path analysis of affecting factors for dough water absorption rate.

| Index | Simple correlation coefficient | | Direct path coefficient |  | Indirect path coefficient | | | | | | | |
| --- | --- | --- | --- | --- | --- | --- | --- | --- | --- | --- | --- | --- |
|  |  |  |  | LMW-GS | Ax1 | Bx7 | Dx5 | Dy10 | ω-gliadin | α/β-gliadin | γ-gliadin | Total |
| LMW-GS | | 0.782 | 0.282 | 0.000 | 0.089 | 0.269 | -0.027 | -0.285 | -0.109 | 0.055 | 0.507 | 0.499 |
| Ax1 | | 0.837 | 0.105 | 0.240 | 0.000 | 0.313 | -0.026 | -0.253 | -0.141 | 0.062 | 0.537 | 0.732 |
| Bx7 | | 0.819 | 0.355 | 0.213 | 0.093 | 0.000 | -0.020 | -0.308 | -0.204 | 0.068 | 0.622 | 0.464 |
| Dx5 | | 0.623 | -0.032 | 0.239 | 0.085 | 0.218 | 0.000 | -0.191 | -0.016 | 0.039 | 0.282 | 0.655 |
| Dy10 | | 0.741 | -0.364 | 0.221 | 0.073 | 0.301 | -0.017 | 0.000 | -0.174 | 0.063 | 0.638 | 1.104 |
| ω-gliadin | | 0.454 | -0.338 | 0.091 | 0.044 | 0.214 | -0.002 | -0.188 | 0.000 | 0.065 | 0.568 | 0.791 |
| α/β-gliadin | | 0.83 | 0.082 | 0.188 | 0.079 | 0.296 | -0.015 | -0.278 | -0.267 | 0.000 | 0.743 | 0.747 |
| γ-gliadin | | 0.856 | 0.796 | 0.180 | 0.071 | 0.277 | -0.011 | -0.292 | -0.241 | 0.077 | 0.000 | 0.060 |

LMW-GS, low-molecular weight glutenin subunits.

**Supplementary Table S9** Path analysis of affecting factors for sedimentation value.

| Index | Simple correlation coefficient | Direct path coefficient |  | Indirect path coefficient | | | | | | | |
| --- | --- | --- | --- | --- | --- | --- | --- | --- | --- | --- | --- |
|  |  |  | LMW-GS | Ax1 | Bx7 | Dx5 | Dy10 | ω-gliadin | α/β-gliadin | γ-gliadin | Total |
| LMW-GS | 0.753 | 0.36 | 0.000 | 0.038 | 0.222 | -0.194 | -0.097 | -0.555 | 0.136 | 0.342 | 0.392 |
| Ax1 | 0.783 | 0.045 | 0.307 | 0.000 | 0.258 | -0.186 | -0.086 | -0.069 | 0.153 | 0.362 | 0.739 |
| Bx7 | 0.848 | 0.293 | 0.273 | 0.040 | 0.000 | -0.140 | -0.105 | -0.100 | 0.169 | 0.419 | 0.555 |
| Dx5 | 0.504 | -0.029 | 0.305 | 0.037 | 0.238 | 0.000 | -0.065 | -0.008 | 0.096 | 0.190 | 0.792 |
| Dy10 | 0.817 | -0.124 | 0.282 | 0.031 | 0.204 | -0.120 | 0.000 | -0.086 | 0.155 | 0.431 | 0.896 |
| ω-gliadin | 0.614 | -0.166 | 0.116 | 0.019 | 0.122 | -0.011 | -0.064 | 0.000 | 0.160 | 0.383 | 0.725 |
| α/β-gliadin | 0.889 | 0.203 | 0.240 | 0.034 | 0.221 | -0.108 | -0.095 | -0.131 | 0.000 | 0.501 | 0.662 |
| γ-gliadin | 0.916 | 0.537 | 0.229 | 0.030 | 0.197 | -0.081 | -0.999 | -0.118 | 0.189 | 0.000 | 0.348 |

LMW-GS, low -molecular weight glutenin subunits.

**Supplementary Table S10** Wheat GSP contents, grain yields, and GSP yields in low and high soil fertility field.

| Soil fertility | Treatment | FDC5 | | |  | BN207 | | |
| --- | --- | --- | --- | --- | --- | --- | --- | --- |
|  |  | GSP  (%) | Yields  (kg/ha) | GSP yields  (kg/ha) |  | GSP (%) | Yields  (kg/ha) | GSP yields  (kg/ha) |
|  |  |  |  |  |  |  |  |  |
| LF | W0N0 | 4.21 | 1470 | 62 |  | 4.67 | 1555 | 73 |
|  | W0N180 | 6.62 | 3455 | 229 |  | 5.24 | 3803 | 199 |
|  | W0N240 | 8.64 | 3872 | 334 |  | 6.46 | 4156 | 268 |
|  | W0N300 | 9.31 | 4149 | 386 |  | 7.77 | 4873 | 379 |
|  | W1N0 | 4.73 | 2619 | 124 |  | 5.00 | 2378 | 119 |
|  | W1N180 | 7.58 | 4481 | 340 |  | 6.90 | 4177 | 288 |
|  | W1N240 | 9.14 | 5103 | 466 |  | 7.79 | 4720 | 368 |
|  | W1N300 | 9.75 | 5338 | 521 |  | 8.40 | 5147 | 432 |
|  | W2N0 | 5.38 | 2708 | 146 |  | 5.31 | 2596 | 138 |
|  | W2N180 | 9.09 | 5058 | 460 |  | 7.89 | 4654 | 367 |
|  | W2N240 | 10.44 | 5846 | 611 |  | 9.05 | 5593 | 506 |
|  | W2N300 | 8.39 | 5187 | 435 |  | 7.71 | 4961 | 382 |
| HF | W0N0 | 4.68 | 4508 | 211 |  | 4.81 | 4044 | 194 |
|  | W0N180 | 7.75 | 6683 | 518 |  | 6.52 | 6953 | 453 |
|  | W0N240 | 8.36 | 7325 | 612 |  | 7.39 | 6988 | 516 |
|  | W0N300 | 8.73 | 7077 | 618 |  | 8.54 | 7126 | 608 |
|  | W1N0 | 5.69 | 5685 | 323 |  | 5.09 | 5068 | 258 |
|  | W1N180 | 7.85 | 9968 | 783 |  | 6.58 | 8194 | 539 |
|  | W1N240 | 8.75 | 9131 | 799 |  | 8.23 | 9411 | 775 |
|  | W1N300 | 8.23 | 9013 | 742 |  | 7.41 | 7216 | 534 |
|  | W2N0 | 4.84 | 6402 | 310 |  | 4.89 | 5858 | 286 |
|  | W2N180 | 6.66 | 10453 | 696 |  | 5.84 | 9805 | 573 |
|  | W2N240 | 7.33 | 9833 | 721 |  | 6.63 | 9610 | 637 |
|  | W2N300 | 7.54 | 9726 | 733 |  | 6.76 | 8294 | 560 |

The values were shown the annual average values of 2018–2019 and 2019–2020. GSP, grain storage proteins; LF, low soil fertility; HF, high soil fertility; FDC5, Fengdecun5; BN207, Bainong207.
